# A Study on the Effects of the Dynamic Features of Light-Based eHMI on Pedestrians’ Crossing Behavior

**DOI:** 10.3390/s26041247

**Published:** 2026-02-14

**Authors:** Yiqi Xiao, Zhiming Liu, Tini Ma, Yingjie Huang

**Affiliations:** 1 College of Publishing, University of Shanghai for Science and Technology, Shanghai 200093, China; yiqi.xiao@usst.edu.cn (Y.X.); 233582932@st.usst.edu.cn (T.M.); 2 School of Design, Royal College of Art Battersea, London SW11 4AY, UK; 10064390@network.rca.ac.uk

**Keywords:** light-based eHMI, yielding intention, headlight, dynamic features, willingness-to-cross

## Abstract

**Highlights:**

**What are the main findings?**

- The research contributes a factorial design framework that systematically isolates the specific effects of animation pattern, speed, and light-emitting area. It identifies animation speed as the most critical dynamic feature of light-based eHMIs, demonstrating that faster light loops significantly deter pedestrians from crossing by signaling non-yielding intent.

**What is the implication of the main finding?**

- The influence of dynamic cues is highly context-dependent, with lighting features significantly affecting decisions primarily during constant vehicle motion or longer time gaps, whereas vehicle kinematics dominate during deceleration. The AV should dynamically adapt lighting characteristics to optimize pedestrian safety in mixed traffic.

**Abstract:**

While light-based external human–machine interfaces (eHMIs) on automated vehicles (AVs) are increasingly studied to mediate pedestrian–vehicle conflicts, gaps persist in understanding how specific dynamic features of the AV’s headlights influence pedestrians’ prediction of its yielding intention and their crossing behavior. This study systematically investigates the effects of dynamic elements of vehicle lighting—including animation patterns, animation speed, and light-emitting area—on pedestrians’ objective and subjective evaluations. A factorial design framework was employed, where participants viewed video simulations of an approaching AV displaying headlight designs combining multiple dynamic features. For different vehicle motion states, the vehicle–pedestrian distance was integrated as a variable to examine its interaction effect with lighting features. Objective measures of cueing effects were complemented by subjective ratings and user preference study via questionnaires. Results showed that there were more crossing behaviors of the pedestrian when presenting higher animation speed of dynamic light eHMIs. Animation pattern and light-emitting area does not play an important role in pedestrian decision-making, but proper design of these two features can evoke higher visual attention. When the vehicle–pedestrian distance is longer, the dynamic features of lighting will more affect people’s willingness to cross. The effects of light eHMIs seemed more significant for the AV travelling in constant speed. Our findings advance preliminary suggestions for selecting light-based eHMIs in the appropriate scenarios and can contribute actionable insights for designing intuitive, human-centric AV–pedestrian negotiation strategies.

## 1. Introduction

Pedestrian–vehicle conflict arises from competing claims to right-of-way between drivers and pedestrians, serving as a critical contributing factor to traffic accidents. Empirical studies indicate that over one-third of drivers fail to yield to pedestrians at marked crosswalks due to factors including higher speeds, increased traffic density, and pedestrian distraction caused by a different activity while crossing [1]. In pedestrian–vehicle conflict scenarios, the strategic interplay between drivers and risk-inducing pedestrians typically resolves through either the driver’s concession or successful deterrence of the pedestrian. During this interplay, there is a process predicated on mutual observation of behavioral cues. Without the driver-centric communication with pedestrians, autonomous vehicles (AVs) cannot socially signal their intentions and decision-making processes to pedestrians whose trajectories intersect theirs. To address this limitation, conceptual AV designs increasingly employ multimodal information that explicitly convey operational intentions and decisions to vulnerable road users (VRUs)–an approach aligned with human habits and preferences [2]. These implementations, collectively termed external human–machine interfaces (eHMIs), have evolved into a distinct research domain supported by a substantial body of related literature.

According to Man et al. [3], eHMI types proposed by researchers to date include icon, light, text, personification, gesture, posture, and color. Among the 38 records included in the systematic review, light-based eHMIs constituted the majority with 21 publications, followed by text-based implementations documented in 11 studies and icon-based solutions appearing in 10 publications. Variants of light-based eHMIs encompass LED light strips, pulsing light bands [4], dynamic light bands [5], and brake light projections [6]. Central design considerations for text-based eHMIs revolve around the selection between ego-centric and vehicle-centric textual presentations [7]. Leveraging users’ prior knowledge, icon-based eHMIs emphasize the appropriate deployment of graphical [8], symbolic [9], and metaphorical icons [10] and pictograms [11] to denote the intention of the vehicle. Exploratory solutions include gaze-interaction systems utilizing eye contact-simulating monitors between pedestrians and AVs [12,13], as well as windshield displays of yielding/non-yielding gestures [14]. Significant research focus has been directed toward evaluating the efficacy of these modalities of explicit information in communicating yielding intentions. In terms of facilitating pedestrian crossing decisions, textual displays demonstrate superior performance as compared to light-based [15] and anthropomorphic eHMI implementations [16]. Text-based interfaces are viewed as offering greater perceived safety and trustworthiness [17], with studies advocating that text is the more preferable display output as it is often intuitive and easy to understand [18]. Combinations of multiple modals of eHMIs have been recommended to enable stage-specific interactions during vehicle motion phases [19,20].

Current research recognizes that eHMIs with unfamiliar and ambiguous information induce more hesitation among pedestrians when encountering vehicles equipped with them. Textual displays on exterior interfaces represent a more language-based communication between pedestrians and vehicles over conventional warning mechanisms like light transitions and horn signals. However, the use of such text-based eHMIs only may present three critical limitations in real-world applications.

First, text-based eHMIs demand greater focused attention from pedestrians as compared to dynamic icons, as evidenced by heightened fixation counts and prolonged fixation durations required for comprehension [21]. Eisma, van Gent and de Winter [22] simulated how pedestrians observe the moving vehicle with their foveal attention and peripheral vision and concluded that the text-based eHMIs captured attention for a longer period than flashing-light eHMIs. When the vehicle is still a certain distance away from the pedestrian, the display of text is not effective enough as the warning sign.

Second, the application of eHMIs in AVs should ideally progress through three sequential stages: aware of situation, perceiving risks, and decision-making [20]. Multiple rounds of information identification require longer response time than implicit communication modalities, and there is not enough time left for VRUs when actual conflicts occur. Almodfer et al. [23] found higher percentages of conflicts over the second and the fourth lane of the two-way and two-lane road towards the crossing direction of pedestrians, as the approaching vehicles on these two lanes frequently fail to detect crossing pedestrians. Under such abrupt conflict scenarios, text-based information requiring semantic interpretation proves ineffective for timely recognition. Even if the vehicle inevitably stops at a distance not far from the pedestrians when they persist in crossing, the pedestrians may not be visually oriented to read the text in a state of panic.

Third, static eHMIs often suffer from directional ambiguity regarding their intended recipients. Hensch, Neumann, and Beggiato [24] suggested that the directedness of steady light signals should be considered when implementing a light-based eHMI in AVs. While dynamic lighting effects can mitigate this issue, such solutions remain incompatible with purely graphical/textual displays that lack inherent motion parameters.

Traditionally, vehicular lighting systems have served as direct communication modalities between drivers and pedestrians or between vehicles, forming established light-based lexicons. These luminous signals not only express human drivers’ intentions regarding imminent maneuvers but also convey implicit information about their emotional states through systematic relationships between luminance parameters and the circumplex model of affect [25]. While novel light patterns remain semantically ambiguous to pedestrians, operational extremes within specific light features can be mapped to dichotomous yielding/non-yielding decisions [26]. This one-to-one mapping enables light-based eHMIs to serve as reliable indicators for rapid pedestrian decision-making scenarios. For AVs, systematic identification of lighting design features that optimally communicate yielding/non-yielding intentionality is essential for enhancing interpretability of vehicular behaviors.

Existing literature evaluating light-based eHMIs documents a variety of light designs. Primarily, the visual characteristics of these luminous interfaces can be categorized into static and dynamic types. Static visual features pertain to fundamental attributes of lighting configurations. Regarding the shape of light, most implementations predominantly employ straight light strips [4,26,27,28,29,30]. Lyu et al. [31] conducted comparative analyses between hexagonal cyan light bands encircling vehicle grilles and alternative designs featuring smiling curves and symbol-based lighting in terms of clarity. Song et al. [32] developed sequential arrow-shaped light strips to indicate acceleration/deceleration patterns. Color is another design element hypothesized to influence pedestrian interpretation of vehicular intent. Aligned with conventional light semantics, red typically denotes stopping while green signals permission to proceed. Several studies have adapted this chromatic coding to eHMI systems, associating green with yielding/safe states and red with non-yielding/unsafe conditions [28,33,34]. Alternative cool-toned hues including cyan [17,35] and turquoise [36] have been frequently compared against green baselines. These researches suggest that color has been a widely recognized visual feature for expressing deceleration intent, i.e., red-colored lights typically indicate that pedestrians should not cross, while cyan-colored lights suggest they may cross.

Current research exhibits limited investigation into the effects of brightness and the lighting area. Carlowitz et al. [37] evaluated two light designs called Full Light Band (FLB) and Partial Light Band (PLB), reporting significant differences regarding the visibility of these two designs. While the relationship between brightness and status indicators in electronic devices has been discussed [38], these findings have not informed AV eHMI design practices. Among dynamic lighting features, research attention has predominantly focused on the direction of light movement. Dey et al. [35] employed crowdsourcing methodology to collect subjective evaluations from 400 participants regarding various laterally-sweeping light animations. Their results found no statistically significant differences in intuitiveness between inward sweeping, outward sweeping, and alternating inward/outward sweeping patterns. Similar investigations have adopted animations featuring light movement from both ends toward the center of the light strip attached to the front grill [28,39,40]. Lim and Kwon [15] supported the superiority of vertical light bands over horizontal ones in conveying urgent warnings. Song et al. [32] demonstrated that rearward-arrow dynamic light strips effectively increase perceived safety for decelerating vehicles, whereas forward-arrow light patterns fail to enhance pedestrian’s perceived safety for accelerating vehicles. Ergonomic comparisons between animation types constitute another research focus, e.g., threads of diodes versus LED light strips [11], flashing versus pulsing lights [40,41], and flashing versus sweeping lights [39,42]. Notably, Lau, Le, and Oehl [43] implemented pulsating LED light bands to indicate vehicular intent while utilizing a light segment that moves with the band to represent the awareness of vehicle. To sum, these studies suggest distinct animation patterns possess unique advantages in alerting the pedestrian.

Lighting systems used in some transportation facilities exhibit additional dynamic characteristics like fluidity and rhythmic regularity, featured by different speeds of the dynamic effects. For instance, fast pulsation of the light was mapped to the acceleration process and slow pulsation to the braking process of the autonomous bus [43]. For a dynamic light, shorter pedestrians’ crossing initiation times and higher trust were detected than for a static light [44,45]. In robotic systems including drones, wheeled robots, and electronic devices, lighting can demonstrate their operational states and forthcoming action plans. Numerous proof-of-concept light behaviors had been selected as the experimental material in related studies [38,46,47] to demonstrate the tasks by improving the expressivity of light. These implementations vary in temporal patterns, with some exhibiting regular pauses while others display inconsistent speed of the pulse frequency or movement periodicity. Empirical evidence suggests constant and smooth brightness profiles are perceived as more pleasant [25]. For robot–human interaction tasks including Alarm [48], Blocked [49,50], and Approaching [46], specific lighting effects contribute to enhanced comprehension of the intention of the agent. The dynamic characteristics underlying these effects can be extracted as the potential for human–vehicle communication-oriented AV headlight design.

Despite beneficial findings having been obtained, current investigations on light-based eHMIs predominantly compare independent light designs while exploring less the origins and generative mechanisms underlying their differences. Our investigation is motivated by the fundamental requirement that the headlight should serve as the medium for communicating real-time decisions of the AVs during dynamic interaction with pedestrians. This proposed dynamic interaction framework necessitates the AV to alternate between yielding and non-yielding states based on real-time distance monitoring and pedestrians’ crossing behavior. Correspondingly, lighting systems can visualize the degree of this binary state through amplification or attenuation of specific dynamic features. When these features effectively encode the intention of AVs, they may surpass conventional flashing signals in conveying richer information for communication. For instance, a specially encoded dynamic feature of the headlight may be regarded as a signal of vehicle acceleration or non-deceleration, whereas its disappearance could indicate impending deceleration. Such designs might be effective eHMI implementations ensuring transparency regarding AVs’ nuanced decision-making processes for VRUs.

Given the lack of robust evidence demonstrating how the dynamic features of AVs’ headlights influence pedestrians’ crossing behavior, we conducted the present study. With this core research question, two specific objectives were formulated:

(1) Objectively measure the cueing effects of representative dynamic features of AVs’ headlights in influencing pedestrians’ crossing behaviors;

(2) Performing subjective evaluations regarding the effectiveness of selected dynamic features of AVs’ headlights in communicating yielding intentions and revealing the attributes contributing to the perception of lighting.

**Figure 1:**
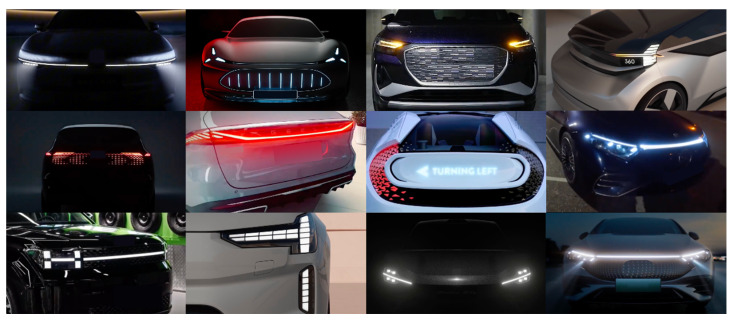
Some lighting designs in the case library.

To identify the features to be studied, we collected 66 lighting designs of automobiles produced in Mainland China, Germany, Japan, and the United States between 2020 and 2025 and supplemented them with 25 lighting concept designs released online during the same period to form a case library (Figure 1). The lighting effect elements of the cases were then extracted and coded individually. We define lighting’s dynamic features as the elements related to the animation of light, which include animation patterns, dimension of the light source, contour and shape of the light, texture and matrix effects, luminous intensity, gradient transitions, movement direction, animation speed, fluidity, rhythmic regularity, flashing duration, etc. Features highlighted in lighting effects used primarily to provide brand image and emotional value were not included in the scope of this study. Ultimately, we determined the luminance, animation pattern, and animation speed of the light as the dynamic features to be investigated.

This study employed a factorial design framework to measure the effects of the predefined dynamic features. The main body of the experiment asked naïve participants to view video simulations depicting a virtual AV approaching head-on while activating the headlight, which presented a combination of multiple representative dynamic features. Participants predicted the vehicular yielding intention reflected through the lighting designs—either willingness or reluctance to yield. Statistical analysis of participants’ response to the lighting could suggest which features more promote pedestrians’ crossing behavior and the decision efficiency of the behavior. Considering the decisive influence of speed reduction of the car on predicting its intention, experimental videos exhibited two vehicular motion cues. Moreover, we simulated varying distances between the vehicle and pedestrian to examine interactive effects between this factor and dynamic features of light eHMIs on pedestrian judgments. Supplementary questionnaires collected subjective ratings of lighting designs containing specific dynamic features. These subjective results and experimental data can corroborate each other about the effect of investigated dynamic features. Section 2 elaborates on experimental design specifics. The aforementioned empirical findings are presented in Section 3 of this paper with a comprehensive discussion in Section 4.

## 2. Materials and Methods

### 2.1. Participants

We recruited college student participants from diverse disciplinary backgrounds, including humanities, natural sciences, engineering, arts, and management, to report their driving experiences, with the age range of 18–25 years old. The numbers of participants from each disciplinary background were approximately equal. Participants were required to self-report normal or corrected-to-normal vision. Subsequently, we employed stratified random sampling method to randomly select experimental participants from the candidate pool, adhering to the principle of equal group sizes for driving-experienced and non-experienced groups. Finally, forty participants were selected (18 males and 22 females; M = 21.65 years old; SD = 1.95).

### 2.2. Apparatus

The effects of light eHMIs were evaluated in a video-based experiment using the paradigm applied in the study of Dey et al. [4,21,51]. The experiment was conducted in a 60-square-meter enclosed laboratory. Experimental equipment included: a 65-inch movable large display with 3840 × 2160-pixel resolution, a laptop installed with Eprime 3.0 software (Psychology Software Tools, Inc., Sharpsburg, PA, USA), and a mini keyboard connected with the laptop. The illumination intensity in the laboratory was maintained at 200 Lx. The position of the display was deliberately adjusted to eliminate glare reflections, ensuring high-fidelity transmission of light properties to participants.

### 2.3. Independent and Dependent Variables

The dynamic features of AVs’ headlights to be evaluated in this study include light-emitting area, animation pattern, and animation speed. We define these features as follows and explain the reasons for selecting them.

#### 2.3.1. Independent Variables

Luminance is defined as the ratio of a light source’s luminous intensity to its projected area. If a single circular light is duplicated to form a light band or an entire array of lights, the light band or array undoubtedly demonstrates higher luminance than the single circular light under the condition of constant luminous intensity of each light unit. In our study, different luminance levels were only determined by the area of light source. Since the number of headlights has been proved to be effective in accurate estimate of motorcycle speed [52], it was hypothesized that difference in the size of the light-emitting area of the headlight may affect individuals’ judgment of AVs’ yielding.

Animation patterns may elicit specific semantic associations, activating participants’ prior experiences related to warning light signals. Overall, the designs of AVs’ light in existing studies primarily fall into two categories: one is the light sweeping along certain trajectories, and the other one is flashing or pulsing. The first animation pattern involved in the experiment is periodically outward sweeping (from middle to the edges of the bumper) of two light segments [35], and the second is rhythmic blink (alternately glows with full brightness and then turns off) of the whole light band [46].

Animation speed refers to the rate of light’s looping animation, which represents its cyclical blinking. From the emotional design perspective, the high frequency of LED change is associated with a restless or unpleasant feeling, and certainly not a signal for a calming and serene mood [25]. The combination of animation speed and pattern may implicitly suggest the probability that the AV will yield through both egocentric (explicit representation of the AV’s states) and allocentric (request to the pedestrian) aspects.

This study also incorporated two crucial independent variables that related to the vehicle’s own motion and would affect pedestrians’ prediction of vehicular intentions. The first one is vehicle motion state. Existing studies [32,53,54,55] have incorporated vehicle motion into experimental designs and confirmed its impact on the signaling efficacy of eHMIs and pedestrians’ risk perception. Relevant findings suggest that when an AV indicates more defensive locomotion maneuvers with the deceleration behaviors having been noticed, it is more likely to be perceived by the pedestrian as safe and prosocial [56,57]. Regarding the threshold value at which speed can have effects, the likelihood of a significantly higher pedestrian head-deviation angle was observed when vehicle speed exceeded 30 km/h [58]. Gould et al. [52] adopts the vehicle speed of 30 mph as a fixed value for judgment of the approaching rate. We implemented two treatments (i.e., constant speed/deceleration) of vehicle motion state. The constant speed was set at 30 km/h. For the deceleration state, the target vehicle decelerated from 30 km/h to 0 km/h at a constant acceleration.

In addition to the lighting effects, another independent variable that could affect pedestrian crossing behavior is time gap [32]. It refers to the time required for the AV to reach the pedestrian’s position, reflecting the current relative pedestrian–vehicle distance. The experiment set 2.5 s and 5 s as the two levels for the time gap.

#### 2.3.2. Dependent Variables

Six dependent variables were measured to analyze pedestrians’ behaviors and their evaluations of making decisions. These include two objective metrics, crossing decision and crossing decision time, and four subjective metrics: perceived likelihood of the AV detecting VRUs, perceived likelihood of vehicular yielding, task difficulty and perceived safety.

Crossing decision refers to whether participants chose to proceed or wait for the vehicle to pass when encountering an approaching AV under specific conditions. It was recorded as binary values (0 or 1), 0 indicating the decision to wait, and 1 indicating the decision to go. Crossing decision time was defined as the interval between participants noticing the lights on the approaching vehicle and making their crossing decision. This is a common measurement in similar studies used to reflect the hesitation of the pedestrian before crossing the road [59].

Willingness-to-cross is a common subjective metric in eHMI-related research. It is a surrogate measure for the pedestrian’s feeling of safety. Walker et al. [60] developed a slider-like potentiometer acting as the input device for the experiment participants to record their judgement in a continuous, real-time manner. Using this device, the experimenters acquired data with two extremities mapped to 0% and 100%, which represents “not willing to cross” and “totally willing to cross” respectively. In the absence of such equipment, we recorded willingness-to-cross using non-continuous equidistant variables. A 10-point Likert scale was used to measure participants’ perceived likelihood of vehicular yielding after the experiment. Normally, if participants believed that light effects under a specific speed indicated yielding, they were more likely to decide to proceed. Therefore, the relationship between the average percentage of this likelihood and crossing decisions can reflect the reliability of the headlights in representing AVs’ intent. If this perceived likelihood is low but crossing decisions are frequent, it suggests the contradiction between the two sets of data; if this perceived likelihood is high but crossing decisions are not so frequent, it implies that the participants actually rate the headlights as not reliable. Similarly, the perceived likelihood of the AV detecting VRUs was also measured using this scale. This likelihood is defined as the percentage estimated by participants that their presence would be successfully detected by the AV.

Among the remaining subjective metrics, task difficulty refers to participants’ self-reported difficulty in judging the AV’s intent based on a specific presentation of light effects at a given motion state or pedestrian–vehicle distance. This metric was set to estimate the overall mental workload required for the task as perceived by the participants, derived and modified from the After-Scenario Questionnaire (ASQ) [61]. Perceived safety [32,42] refers to the participants’ sense of security for the presented road-crossing opportunity. These three metrics are marked by the participants on the 7-point Likert scale.

### 2.4. Experimental Setup

We implemented a within-subjects experimental design, with two blocks in a four-factor (2 light-emitting area × 2 animation pattern × 2 animation speed × 2 time gap = 16 combinations) full factorial design. Each participant was required to decide to cross the road or not when presented with a combination of the three attributes of light. Each of the eight combinations constituted a distinctive eHMI type. Vehicle motion state was set as a between-block variable. In one block, the target AV approached at a constant speed; in the other it decelerated until fully stopping.

**Figure 2:**
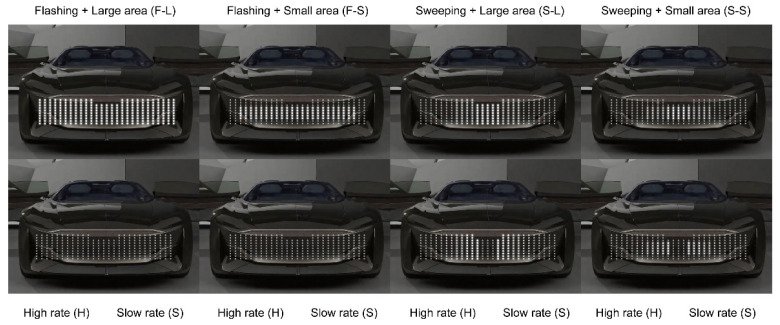
Eight eHMI types combining the three dynamic features. F-L-H is the abbreviation of flashing light (F) with large light-emitting area (L) and high rate of animation loop (H); see the first column from left. The second column depicts the changing process of the lighting effect through another animation frame; the speed of this process has two levels: fast and slow. The other eHMI types are also abbreviated in this manner. Abbreviations are not applicable for two treatments of time gap.

**Figure 3:**
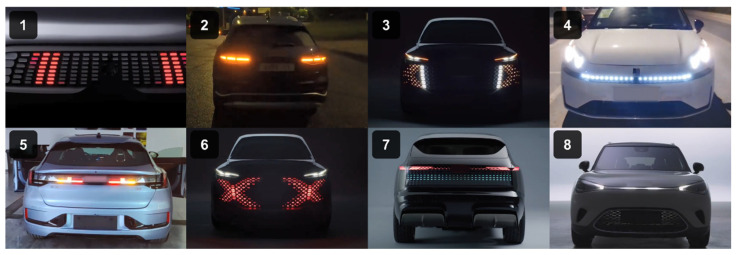
Eight eHMI types in real car design (No. 1: S-L-H; No. 2: S-S-H; No. 3: S-L-S; No. 4: S-S-S; No. 5: F-S-H; No. 6: F-L-H; No. 7: F-L-S; No. 8: F-S-S).

To measure the impact of display positions, some studies have positioned light-based eHMIs on multiple vehicle components, involving the roof, windscreen, and grille [62], and compared their effectiveness in signaling. To reduce participants’ mental workload related to visual attention and focus on measuring the effects of lighting dynamic features, we investigated only the design elements of the headlights. For the size of the light-emitting area, two treatments were applied: a smaller one and another twice as large. Regarding the animation pattern, sweeping along certain trajectories features a light movement from the center toward both ends of the lighting area, while flashing is regular rhythmic blinking. Two levels of animation speed were set for the experiment. One loop for the high rate of the looping animations lasted 250 ms with 20 repetitions, and one for the slow rate lasted 500 ms with 10 repetitions (Figure 2). Figure 3 depicted the application of the eight eHMI types to car design.

**Figure 4:**
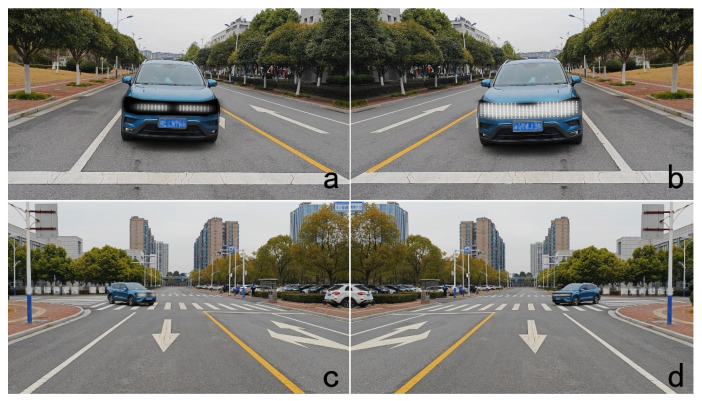
The two views of an approaching AV. (**a**,**b**) an AV is approaching from the pedestrian’s left and right side; (**c**,**d**) an AV is approaching from the pedestrian’s left front followed by a right turn and from the right front followed by a left turn.

We captured raw daytime video clips of an approaching Volkswagen SUV with a black mask applied to its grille part to simulate the area of the headlight. The visualizations of the light eHMI were added with After Effect 2024 software (Adobe Systems Incorporated, San Jose, CA, USA). By editing the videos, the light effects to be tested were overlaid on this mask, as shown in Figure 2. The color of the light effect for testing was white (RGB: 255, 255, 255). When seeing the videos, the pedestrian should imagine that the current location was at the curbside of a straight road. To mitigate the effects of the outliers, each combination was tested with two repetitions in which the AV approached from different views. We proposed two fundamental views [63]: the first involved the AV approaching from the pedestrian’s left or right side while the pedestrian prepares to cross forward at a crosswalk without the signal light; the second involved the AV approaching from the pedestrian’s left or right front, followed by a left or right turn, while the pedestrian is crossing forward not at the crosswalk (Figure 4). For both of the two fundamental views, videos were displayed to the participant in landscape orientation. The frequency of presenting two opposite orientations of the AV’s approach was the same. All the 16 combinations were displayed in a randomized order to prevent learning effects for each block. The two views for one combination were also randomly presented. Furthermore, the order of testing the two blocks was counterbalanced across participants. Every participant completed 64 trials in total (8 eHMI type × 2 time gap × 2 repetition × 2 vehicle motion state).

In the video, the AV’s starting position was adjusted for two time gaps. When the AV travelled at a constant speed for 5 s, the pedestrian–vehicle distance was approximately 42 m, while it was 21 m for 2.5 s. Whenever the AV approached with or without deceleration, it would stop 2.5 m in front of the pedestrian in the reality. For the AV approaching in constant speed, its stop was abrupt. The physical size of the AV’s headlight displayed on the screen in the last frame was 48 × 9 cm for both treatments of speed.

### 2.5. Procedures

The experiment aimed to investigate pedestrians’ willingness-to-cross after exposure to different light-based cues. Prior to the formal experiment, each participant was briefed on the experiment’s objectives. A pre-experiment consisting of 10 practice trials was prepared for the participants to familiarize themselves with the experimental setup and comprehend the objectives. The pre-experiment employed an identical apparatus and setup to the formal experiment, except that the presentation of eight distinct light-based eHMIs was replaced with three alternative light effects. Each of these three light effects incorporated all the three dynamic features. This design ensured that participants became acquainted with the formal experiment’s procedure while avoiding the learning effect before they first encountered the experimental light-based eHMIs. Participants were advised to express their immediate judgments instead of hesitating to cross in the trials to guess the lights’ meanings, as there are no specific associations between light and vehicle behavior. They definitely retained the right to withdraw if they felt unable to perform the required experiment tasks for any reason. Those who agreed to proceed signed a consent form. Participant recruitment criteria and the consent form were reviewed and approved by the ethical review committee at the authors’ institution.

The formal experiment consisted of 64 trials and a subjective questionnaire phase. To avoid carry-out effects, after completing the 8 trials for one eHMI type (2 time gap × 2 repetition × 2 vehicle motion state), the test for the next eHMI type was conducted one week later. The whole experiment lasted for 7 weeks. The order of exposure of eHMI types was counterbalanced across participants. The participants were not informed of the differences in dynamic feature between the presented eHMIs in each weekly test session. Finally, the decisions made by the participants in each week would be statistically analyzed to determine whether they developed a certain response strategy towards specific lighting elements.

**Figure 5:**
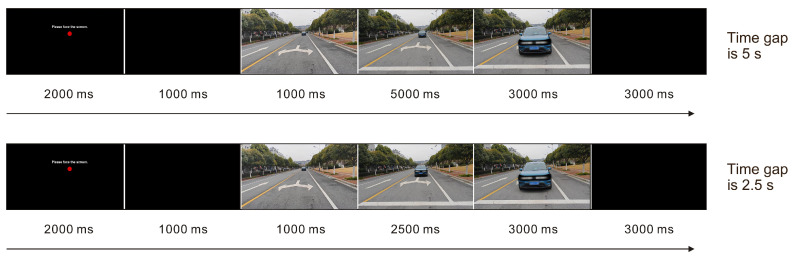
The timeline of experimental procedure.

Due to the different vehicle speeds and pedestrian–vehicle distances set, the experimental trials exhibited four different durations. For the approach taking 5 s with constant speed, a single trial in the formal experiment lasted 15 s (Figure 5), divided into the priming phase and the testing phase. When the priming phase was initiated, a small red dot was presented on the screen for two seconds to indicate the position where the AV would appear, followed by a one-second black frame. Participants were instructed to pay attention to the appearance of the red dot and immediately watch for vehicles approaching from the side after the street scene appeared. Once the light was displayed, the testing phase initiated. The participant would see the AV’s appearance at its beginning, followed by activation of the headlight after one second. If the participant decided to cross, he or she should take the corresponding action by pressing the Up key on the keyboard. The state of the vehicle being fully stopped was shown for 3 s. After that, there was a three-second black frame acting as the interval before the next clip. If the participant could not make a decision within the duration from lighting to the full stop (i.e., the time gap), the trial was deemed invalid and its data were excluded. The timeline of the approach taking 2.5 s is illustrated in Figure 5.

The subjective questionnaire phase was initiated after the test of one eHMI type per week. The participants reviewed the videos of this eHMI for two vehicle motion states. Then, they first rated the eHMI for two time gaps in the perceived likelihood the AV had detected VRUs and the likelihood of yielding under the constant speed condition (Table 1a). Subsequently, they rated it for two time gaps in task difficulty and perceived safety when the AV would gradually decelerate and would not, using the questionnaire in Table 1b. Finally, participants were asked to review their crossing decisions for this eHMI under the two motion states and then provide oral explanations. Upon completing the experiment, each participant received a compensation of 25 yuan.

### 2.6. Data Processing

We employed chi-square tests to analyze the effects of the independent variables on pedestrians’ crossing decisions, under the assumption that pedestrians would never compete with the AV for right-of-way. We ranked all the eight eHMI types based on the frequency of decision-to-cross. The data of crossing decision time were processed using repeated-measures ANOVA to identify main effects and interaction effects related to the independent variables. Mann–Whitney’s U test was applied to detect statistical differences in the scores of subjective metrics for the independent variables. In order to compare the eHMI types in these metrics, Kruskal–Wallis H test was performed. All objective and subjective data were calculated using SPSS Statistics 27.0 software. For statistics of user preference study, descriptive statistics were used to identify users’ top preference. All verbal explanations of subjective scores and user preferences were audio-recorded and subsequently verbatim transcribed to extract significant responses or critical themes.

## 3. Results

### 3.1. Objective Measures

Objective measures differed between the two blocks where the AV was traveling at a constant speed versus decelerating; the relevant experimental results for each are reported separately in this Section. To minimize potential learning effects, we firstly examined changes in participants’ crossing decisions across weeks when facing large/small areas and high/slow looping of animation. The results revealed that the frequency of non-crossing decisions for large areas (χ^2^ = 4.54, *p* = 2.09) and high looping rates (χ^2^ = 0.33, *p* = 0.95) remained stable over time, as did the frequency of crossing decisions for small areas (χ^2^ = 0.54, *p* = 0.91) with low looping rates (χ^2^ = 1.09, *p* = 0.78).

#### 3.1.1. The State Where the AV Is Travelling at a Constant Speed

**Figure 6:**
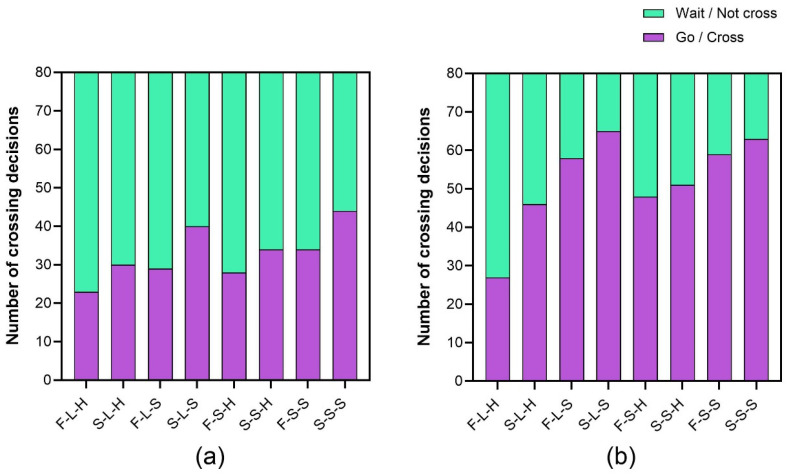
The distributions of crossing decisions of the 8 eHMI types for 2.5 s (**a**) and 5 s (**b**) when the AV moved at a constant speed.

Statistical analysis of crossing decision frequency revealed significant effects of light-emitting area (χ^2^ = 5.80, *p* < 0.05, φ = −0.07), animation pattern (χ^2^ = 14.08, *p* < 0.001, φ = −0.11), animation speed (χ^2^ = 34.58, *p* < 0.001, φ = −0.16), and time gap (χ^2^ = 75.36, *p* < 0.001, φ = −0.24) on pedestrians’ crossing behavior. Time gap exhibited the strongest effect, followed by animation speed, animation pattern, and headlight brightness. Obviously, the number of decisions not to cross when the time gap was 2.5 s (262 of 640 trials) was much less than 5 s (417 of 640 trials). Regarding animation speed, a higher rate of animation loops appeared to more strongly prompt pedestrians to avoid crossing in front of the AV (353 of 640 trials) than slower loops (248 of 640 trials). When the AV displayed rhythmic blinking, the proportion of trials where pedestrians tended to wait and yield (334 of 640 trials) exceeded that when dynamic outward sweeping lights were presented (267 of 640 trials). When the size of the headlight doubled and projected a brighter light, the proportion of trials with pedestrian waiting behavior (322 of 640 trials) was higher than a less bright light (279 of 640 trials). Overall, when facing an AV approaching at a moderately constant speed and signaling with lighting effects, pedestrians slightly leaned toward proceeding/crossing (n = 659) rather than waiting/not crossing (n = 601). Frequencies of crossing decisions for all the 16 combinations are shown in Figure 6.

Excluding time gap as an independent variable, we compared the crossing decision proportions of the eight eHMI types. The ascending order of crossing decision proportions by eHMI type was: F-L-H (31.25%), F-S-H (47.50%), S-L-H (47.50%), S-S-H (53.13%), F-L-S (54.38%), F-S-S (58.13%), S-L-S (65.63%), and S-S-S (66.88%). The distribution of crossing decisions exhibited statistically significant differences across the eight eHMI types (χ^2^ = 58.69, *p* < 0.001, φ = 0.21).

Given the substantial impact of time gap on pedestrian crossing decisions, we further examined the decision frequencies for the eight eHMI types at the same gap. Results indicated statistically significant differences in the distribution of crossing decisions across the eight eHMI types regardless of whether the AV approached within 2.5 s (χ^2^ = 16.62, *p* < 0.05, φ = 0.16) or 5 s (χ^2^ = 57.97, *p* < 0.001, φ = 0.30). When the AV approached within 2.5 s, the S-S-S configuration elicited proceed/crossing in 44/80 trials (55%), making it the only eHMI type with crossing decisions over 50 percent. When the AV approached within 5 s, only the F-L-H configuration resulted in fewer than 50% proceed/crossing decisions (n = 27/80). It can be inferred that the light-based eHMI using F-L-H configuration was the most superior among the eight types in suppressing pedestrians’ crossing behaviors if the AV was not intended to yield, whereas S-S-S was the worst. For the same time gap, all the features exerted significant effect on crossing decision except light-emitting area under 5 s conditions (χ^2^ = 2.09, *p* = 0.19, φ = 0.06).

**Figure 7:**
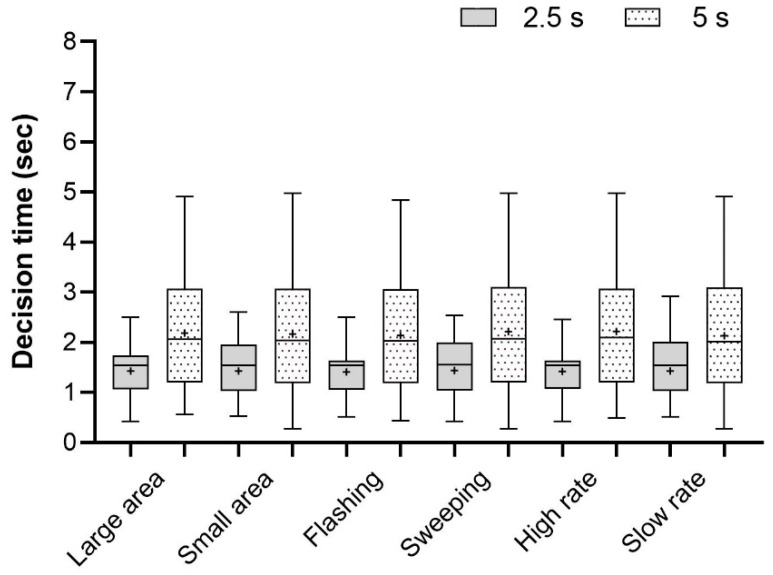
Crossing decision time for constant speed (the + represents the average value).

Apart from time gap (F_(1,1278)_ = 232.30, *p* < 0.001), we detected no statistically significant effects on crossing decision time for any of the other three independent variables (light-emitting area: F_(1,1278)_ = 0.16, *p* = 0.90; animation pattern: F_(1,1278)_ = 0.76, *p* = 0.38; animation speed: F_(1,1278)_ = 0.49, *p* = 0.49). These three features had no statistically significant interaction effects with time gap. Furthermore, no statistically significant differences in decision time emerged across the eight eHMI types (F_(7,1272)_ = 0.25, *p* = 0.97). Whenever the time gap was small (F_(7,632)_ = 0.51, *p* = 0.83) or large (F_(7,632)_ = 0.33, *p* = 0.94), no eHMI elicited significantly shorter crossing decision time than others. Among all the eHMI types, relatively short decision times were observed for F-S-S (M = 1.74 s, SD = 0.99) and F-L-S (M = 1.75 s, SD = 0.93), while longer times occurred for S-L-H (M = 1.86 s, SD = 0.93). Descriptive statistics of the crossing decision time for the independent variables is shown in Figure 7.

#### 3.1.2. The State Where the AV Is Decelerating

A chi-square test was conducted to examine the effects of dynamic features on the occurrence of crossing decision. It showed that the effect of animation speed (χ^2^ = 19.63, *p* < 0.001, φ = 0.12) was statistically significant when the AV is decelerating. When facing the high rate of animation loops, participants were more inclined not to cross the road (93 of 640 trials) compared to the slow rate (44 of 640 trials). This result is similar to that under the constant speed condition. We found no significant effects of light-emitting area (χ^2^ = 0.20, *p* = 0.65, φ = 0.01) and animation pattern (χ^2^ = 0.40, *p* = 0.53, φ = 0.18) on crossing decisions. Overall, the 8 eHMIs (χ^2^ = 20.59, *p* < 0.01, φ = 0.13) resulted in significantly different numbers of crossing decisions. The ascending order of crossing decision proportions by eHMI type was: F-L-H (84.37%), S-L-H (85.62%), S-S-H (85.62%), F-S-H (86.25%), F-L-S (91.87%), F-S-S (92.50%), S-L-S (93.75%), and S-S-S (94.37%).

**Figure 8:**
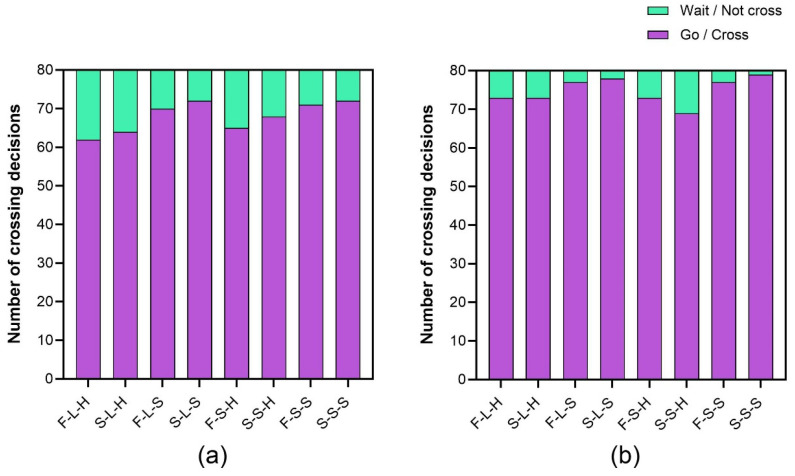
The distributions of crossing decisions of the 8 eHMI types for 2.5 s (**a**) and 5 s (**b**) when the AV decelerated.

Using chi-square tests, we calculated the number of crossing decisions under different pedestrian–vehicle distances. When the AV began to decelerate and signaled with lights at a time gap of 5 s, the number of pedestrians deciding to cross (599 of 640 trials) was higher than at 2.5 s (544 of 640 trials). The effect of animation speed was significant at both time gaps (2.5 s: χ^2^ = 8.28, *p* < 0.01, φ = 0.11; 5 s: χ^2^ = 13.79, *p* < 0.001, φ = 0.15). When the time gap was 5 s, the effect of presenting different eHMI types on the number of crossing decisions was statistically significant (χ^2^ = 16.86, *p* < 0.05, φ = 0.16). Among them, the occurrence of crossing decision for S-S-S is the most frequent (98.8%). Whereas when the time gap was 2.5 s, the difference in eHMI types did not lead to significant differences in the number of crossing decisions. The proportion of crossing decision for the 16 combinations is shown in Figure 8.

**Figure 9:**
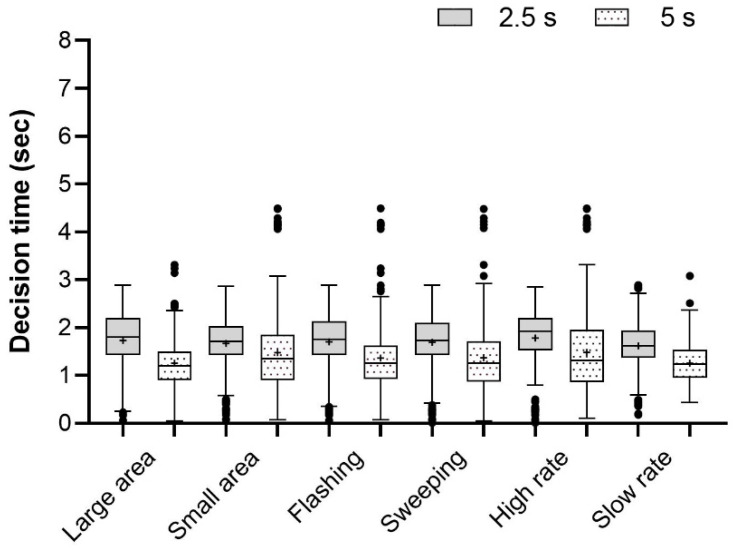
Crossing decision time for deceleration (the + represents the average value).

Statistically significant effects on crossing decision time for animation speed (F_(1,1278)_ = 30.93, *p* < 0.001), light-emitting area (F_(1,1278)_ = 4.96, *p* < 0.05) and time gap (F_(1,1278)_ = 91.82, *p* < 0.001) were found. The effect of animation pattern was minimal (F_(1,1278)_ = 0.00, *p* = 0.96). The difference in decision time across the eight eHMI types was statistically significant (F_(7,1272)_ = 5.29, *p* < 0.001). When the time gap was 2.5 s, a high rate of animation loop resulted in significantly longer decision time than a slow rate (F_(1,638)_ = 15.12, *p* < 0.001). When the time gap was 5 s, a higher rate of animation loop (F_(1,638)_ = 18.09, *p* < 0.001) and smaller light-emitting area (F_(1,638)_ = 17.70, *p* < 0.001) required significantly longer decision time. Among all the eHMI types, relatively short decision times were observed for S-L-S (M = 1.39 s, SD = 0.52), while longer times occurred for F-L-H (M = 1.59 s, SD = 0.65) and S-L-H (M = 1.57 s, SD = 0.65). Descriptive statistics of the crossing decision time for the independent variables is shown in Figure 9.

### 3.2. Subjective Ratings

#### 3.2.1. Measures for the State Where the AV Is Travelling at a Constant Speed

**Figure 10:**
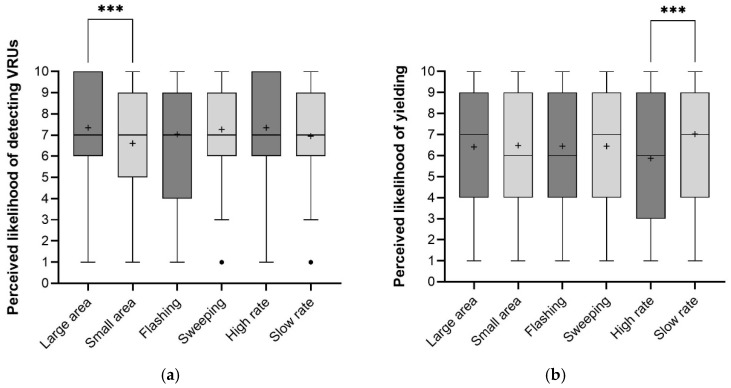
Descriptive statistics of the perceived likelihood of detecting VRUs (**a**) and yielding (**b**) (the + represents the average value; *** indicates significance at *p* < 0.001).

Regarding the perceived likelihood of the AV detecting VRUs, a statistically significant difference was found between the two light-emitting areas of light-based eHMI (Z = −3.31, *p* = 0.001). For the large area, the mean likelihood rating (M = 73.44%, SD = 2.55) exceeded that for the small area (M = 66.06%, SD = 2.19). Participants’ estimates of the likelihood that the AV detected VRUs varied significantly across two time gaps (Z = −5.60, *p* < 0.001). In contrast, neither animation pattern (Z = −1.08, *p* = 0.28) nor animation speed (Z = −0.66, *p* = 0.51) showed statistically significant differences for this rating (Figure 10a). We found no statistically significant difference in this rating across the eight eHMI types (Kruskal–Wallis H test: χ^2^ = 13.74, df = 7, *p* = 0.06). The F-L-S yielded the highest average rating among all eHMIs (M = 77.50%, SD = 2.08), while S-S-S yielded the lowest (M = 62.75%, SD = 2.33). Among the 16 combinations, the highest mean score occurred when the AV approached slowly with the F-L-S configuration (M = 87.00%, SD = 1.75), and the lowest occurred when the AV approached rapidly with the S-S-S configuration (M = 53.50%, SD = 1.90).

There was a statistically significant difference in participants’ perceived likelihood of vehicular yielding between two time gaps (Z = −9.13, *p* < 0.001). When the animation loop was at a high rate, participants’ estimated likelihood of vehicular yielding (M = 60.06%, SD = 2.88) was significantly lower (Z = −3.59, *p* < 0.001) than at a slow rate (M = 68.04%, SD = 2.57). Neither animation pattern (Z = −0.02, *p* = 0.98) nor light-emitting area (Z = −0.15, *p* = 0.88) showed statistically significant differences in these ratings (Figure 10b). The Kruskal–Wallis H test revealed that the eight eHMI types differed in this metric significantly (χ^2^ = 14.11, df = 7, *p* < 0.05). Among these types, F-S-S yielded the highest mean score (M = 73.50%, SD = 2.44), while F-S-H (M = 57.00%, SD = 2.95) and F-L-H (M = 57.50%, SD = 3.07) both scored low. Among the 16 combinations, presenting S-S-H when the AV approached within 5 s was perceived as most indicative of yielding intent (M = 87.00%, SD = 1.75), whereas presenting F-L-H when the AV approached within 2.5 s (M = 41.00%, SD = 2.38) most poorly conveyed this intent.

Simple effect analyses of the three dynamic features under the same time gap were performed. For perceived likelihood of the AV detecting VRUs: large light-emitting area > small area at 5 s (Z = −3.18, *p* = 0.001). For perceived likelihood of vehicular yielding: slow rate of animation loop > high rate at both 2.5 s (Z = −3.17, *p* < 0.01) and 5 s (Z = −2.74, *p* < 0.01).

#### 3.2.2. Measures for the Two Vehicle Motion States

**Figure 11:**
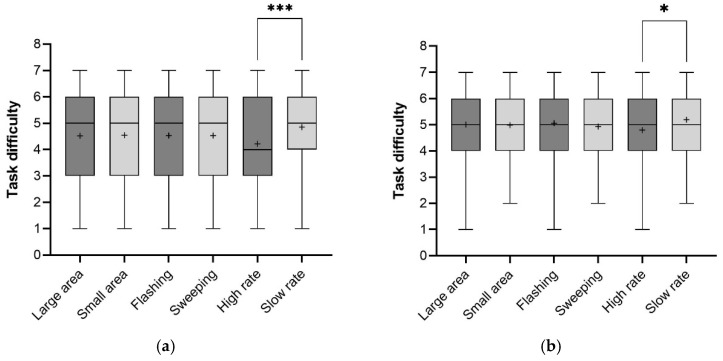
Descriptive statistics of task difficulty for constant speed (**a**) and deceleration (**b**) (the + represents the average value; *** indicates significance at *p* < 0.001, * indicates significance at *p* < 0.05).

**Figure 12:**
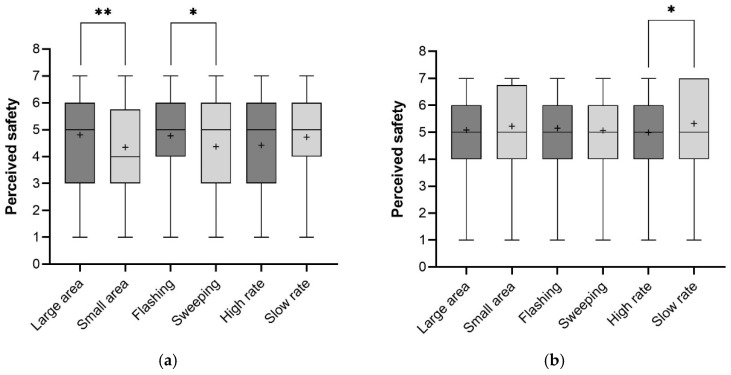
Descriptive statistics of perceived safety for constant speed (**a**) and deceleration (**b**) (the + represents the average value; ** indicates significance at *p* < 0.01, * indicates significance at *p* < 0.05).

The averaged task difficulty for each feature under two motion states is plotted in Figure 11a,b. Figure 12a,b depict the average ratings across the eight eHMI types in perceived safety.

(1) The AV is travelling at a constant speed

Although the effects of animation speed on crossing decision time were not demonstrated, they showed a significant effect on task difficulty ratings (Z = −3.35, *p* = 0.001), as did time gap (Z = −8.55, *p* < 0.001). Notably, a high rate of animation loop yielded higher perceived task difficulty (M = 4.21, SD = 1.68) than a slow rate (M = 4.85, SD = 1.70), suggesting rapid changes of lighting may increase pedestrians’ hesitation. This aligned with the finding that faster looping animations resulted in fewer crossing decisions. Effects of light-emitting area (Z = −0.09, *p* = 0.93) and animation pattern (Z = −0.003, *p* = 0.99) on task difficulty ratings were minimal. The F-L-S configuration received the highest average rating among the eight eHMI types (M = 4.93, SD = 1.77), while F-L-H received the lowest (M = 4.15, SD = 1.76). Overall, no statistically significant differences were detected across eHMIs for task difficulty (Kruskal–Wallis H test: χ^2^ = 11.54, df = 7, *p* = 0.12). Simple effect analyses showed that a high rate of animation loop was higher rated in task difficulty than a slow rate at 5 s (Z = −4.38, *p* < 0.001).

Statistical data indicated significant effects of light-emitting area (Z = −2.60, *p* < 0.01), animation pattern (Z = −2.00, *p* < 0.05), and time gap (Z = −5.04, *p* < 0.001) on perceived safety. The effect of animation speed (high rate: M = 4.81, SD = 1.79; slow rate: M = 4.34, SD = 1.61) was notable but not statistically significant (Z = −1.44, *p* = 0.16). The results demonstrated that light-emitting area and animation pattern were perceived as more influential for the pedestrian to estimate the safety among the three dynamic features. Statistical differences in ratings of perceived safety across the eight eHMI types were significant (Kruskal–Wallis H test: χ^2^ = 14.73, df = 7, *p* < 0.05), with F-L-S yielding the highest mean score (M = 5.08, SD = 1.64) and S-S-H the lowest (M = 3.85, SD = 1.64).

Simple effect analyses showed that a high rate of animation loop was higher rated in task difficulty than a slow rate at 5 s (Z = −4.38, *p* < 0.001). Simple effect analyses showed that a small light-emitting area was higher rated in perceived safety than a small area at 5 s (Z = −2.47, *p* < 0.05).

(2) The AV is decelerating

Mann–Whitney’s U test was conducted to measure the effects of dynamic features on task difficulty. Statistical analysis revealed that the subjective scores were not significantly different between two light-emitting areas (Z = −0.21, *p* = 0.83) and two animation patterns (Z = −0.99, *p* = 0.32). The effects of time gap (Z = −8.34, *p* < 0.001) and animation speed (Z = −2.46, *p* < 0.05) were statistically significant. There were no significantly different ratings across the eight eHMI types (Kruskal–Wallis H test: χ^2^ = 7.62, df = 7, *p* = 0.37), with F-S-S (M = 5.30, SD = 1.36) and F-L-S (M = 5.30, SD = 1.38) yielding the highest mean score and S-S-H the lowest (M = 4.73, SD = 1.30). When the time gap was 2.5 s, a high rate of animation loop was rated significantly less satisfying in easily making a crossing decision than a slow rate (Z = −2.02, *p* < 0.05). Other statistically significant interactions between dynamic feature and time gap were not found.

For the perceived safety, it was found that the effects of light-emitting area (Z = −0.93, *p* = 0.35) and animation pattern (Z = −1.04, *p* = 0.30) were not statistically significant. Animation speed (Z = −2.18, *p* < 0.05) had a statistically significant effect, and so did time gap (Z = −4.59, *p* < 0.001). The ratings across the eight eHMI types were not significantly different (Kruskal–Wallis H test: χ^2^ = 10.05, df = 7, *p* = 0.19), with F-S-S (M = 5.67, SD = 1.38) yielding the highest mean score and S-S-H the lowest (M = 4.87, SD = 1.56). When the time gap was 2.5 s, a high rate of animation loop was rated significantly less satisfying in safety perception than a slow rate (Z = −2.29, *p* < 0.05). We did not find other statistically significant interactions between dynamic feature and time gap.

## 4. Discussion

### 4.1. General Discussions

This study aimed to investigate the impact of several fundamental dynamic features of light-based eHMI on pedestrians’ crossing behavior when applied to AV headlights. The research found that among the selected three dynamic features—animation speed, animation pattern, and light-emitting area—animation speed influenced the most metrics. Comparatively, animation speed of the light-based eHMI played a more critical role in pedestrians’ judgment of the automated vehicle’s intent. For the two vehicle motion states, the faster animation loop of band-like headlights resulted in more decisions of not crossing in front of the AV as compared to slower looping. Although no statistically significant effects were demonstrated on crossing decision time, a faster animation loop correlated with lower perceived likelihood of vehicular yielding, indicating that it was more likely interpreted as signals of the vehicle’s unwillingness to yield. Additionally, a faster animation loop increased the perceived difficulty of making crossing decisions, which may reinforce pedestrians’ waiting tendencies. This was also corroborated by the rating of safety in which faster animation looping was more often viewed as a sign of unsafety. It is considered that light-based eHMIs with a higher rate of animation loop may generally more deter pedestrians from crossing the road.

This deterrent effect arises from two causes. First, a high rate of animation loop may indicate that the vehicle is in a specific state, signifying urgent driving demands, abnormal driving maneuvers, or warnings of vehicle hardware damage. For ordinary individuals, a sudden animation with a rhythm markedly faster than normal dynamic lighting could be more or less associated with these phenomena. This assertion is substantiated by two representative quotes from participants. (1) “As an experienced driver, I frequently flash my headlights rapidly and repeatedly to urge the vehicle ahead to speed up when desiring it to.” (2) “I have observed vehicle lights presented with abnormal frequency or color, or at a special position, that are designed to automatically alert external road users to the driver’s incorrect operation; I believe they serve safety warning purposes.” Second, the light animations being suddenly accelerated are more readily perceived as immediate warnings to pedestrians. More than half of participants recalled their experiences with vehicles of special functions (e.g., police cars, ambulances), noting that accelerated rhythms of sirens and warning lights convey escalating urgency. In such scenarios, whether driving or walking, they therefore became more inclined to yield to vehicles emitting such signals. Our results demonstrate that high animation speed can leverage its strong warning nature to urge pedestrian vigilance, yet it is inadequate to communicate explicit action semantics to the VRUs.

The hypothesis that animation pattern influences pedestrians’ crossing decisions was supported only by partial results. When the AV displayed periodically flashing lights, the proportion of trials where pedestrians tended to wait and yield to the vehicle was significantly higher than when lights sweeping from the middle to the edges of the bumper were presented. When the AV travelled in constant speed, participants rated periodically flashing lights as having higher perceived safety. These findings suggested that periodic flashing could perhaps effectively communicate the vehicle’s non-yielding intent under specific conditions. However, animation pattern influenced fewer dependent variables than the other two dynamic features. Neither of the two representative animation patterns exhibited statistically significant differences in either the perceived likelihood of the AV detecting VRUs or the estimated probability of the AV exhibiting yielding intent. This aligned with the conclusions of Dey et al. [35]. They found that color overrode animation patterns in the intuitiveness of lighting under different conditions. Compared to color, animation patterns made a less significant conscious impact on pedestrians’ decision-making. They also noted that uniformly changing patterns were preferable over the moving or sweeping patterns, which also resonated with the findings of this study. Related studies had also confirmed that there was not much difference in people’s acceptance of different forms of light-based eHMI [11].

As a visual element of traffic signals, light-emitting area may function similarly to color in terms of visibility. Consequently, lights with larger light-emitting areas and higher visibility [37] were more likely to be perceived as indicating AVs’ awareness of the VRUs and attempts to alert them. To some extent, this perception inclined individuals to interpret the vehicle as expressing yielding intent. However, solely increasing the light-emitting area does not convey clear semantics of intention that is conducive to the individual’s judgment. Eisma, van Gent and Winter [22] demonstrated that presenting flash eHMIs on large windows did not result in fewer reaction times or lower visual workload for pedestrians as compared to small windows. Furthermore, our experimental results also showed that a small light-emitting area actually prolonged decision time when the AV was decelerating for 5 s. In other words, fewer bright lights caused the pedestrian to waver in judging the intent of an AV decelerating from a far location. We infer that light-emitting area plays only an indirect role in communicating vehicle intent. According to the findings, we recommend employing the light effects with a large area of light source and high brightness when the pedestrian–vehicle distance is relatively long, thereby better attracting their attention.

Empirical data indicated that, in terms of main effects, the expressiveness of lighting could be less useful in traffic scenarios than anticipated. Products with lighting design are predominantly found in smart home devices, robots, and drones, adopting diverse lighting patterns, speeds, and positions from which the light appears. To express their specific functions, using light languages that inform the users of the tasks’ meaning and the current state often serves as a design strategy [38,46,47,50,64]. Nevertheless, there is a distinction between pedestrian–vehicle conflict and functional expression. During a pedestrian–vehicle conflict, the intentions of both the pedestrian and the vehicle will change with real-time situations, whereas appropriate functional expression merely requires identifying a symbolically optimal animation pattern. The efficient approach for an AV to communicate with the external road users is not semantically decoding its lighting, but rather emitting more apparent and intense signals with the lighting to heighten their awareness of potential risks. The lights sweeping from the middle to the edges of the bumper may be more interpretable with symbols and metaphors, yet their visibility may be inferior to rhythmic blinking.

The significant impacts of motion state and time gap are worthy of discussion. In relevant studies, a virtually or physically presented AV decelerated earlier [44] or came to a complete stop in front of the pedestrian to demonstrate its yielding [4,31,37]. For the non-yielding control group, the AV ultimately stopped at the pedestrian’s location. We studied two motion states based on this experimental paradigm. The results indicating no difference in reaction times under constant speed suggest that the dynamic features of the AV’s lighting do not constitute contributing factors to the efficiency of predicting its intention. In contrast, during deceleration, fast animations and large-area lights significantly prolonged decision time, indicating that their appearance caused pedestrians to seek further confirmation of the vehicle’s credibility in continuing to decelerate. Compared to constant speed, the design of the headlights has less impact on crossing decisions during deceleration. Clearly, a visibly decelerating vehicle speed is the most intuitive and reliable cue for making crossing decisions. At this point, high-speed looping lights can make pedestrians worry that the vehicle might not stop at its current rate, whereas merely changing the lighting pattern may not have this effect. This suggests that fast light animations should not be presented with deceleration, as this creates a mismatch in speed between the two signals and leads to misunderstanding. The larger the time gap, the more likely pedestrians are to start crossing at the still-safe situation. If the time gap is too short, the effect of lighting is minimal; for a 2.5 s time gap, no effect of dynamic features was observed on most metrics, whereas some effects only became apparent at 5 s. This indicates that at riskier pedestrian–vehicle distances, pedestrians are less likely to calmly integrate lighting information to predict vehicle behavior, relying instead more on observing the vehicle’s speed.

We recommend appropriate use of the lighting design based on different levels of vehicle speed. When the vehicle–pedestrian distance is close, there is not much reaction time left for pedestrians, therefore they will be more nervous and hesitant in this situation, for which the variations in dynamic features of vehicle light have minimal influence on their perceptions and the subsequent decisions. According to the experimental results, increasing the rate of animation loop may be the only viable approach to warn pedestrians from a distance. When the vehicle travels at a low speed, enhancing brightness of the light-based eHMI through enlarging the light-emitting area could compensate for diminished perceived risk induced by slower vehicle speeds. If the AV intends to yield, employing the lighting with a slow rate of animation loop is more recommended to improve pedestrians’ safety perception and decision efficiency.

By reviewing the objective results, subjective evaluations, and user-preferred lighting designs, it was found that the frontal brake light [27] using the flashing (rhythmic blinking) pattern with a large light-emitting area and high rate of animation loop outperformed in pre-warning pedestrians of the risks of the AV and in restraining them from crossing. If the AV decides to yield, this lighting effect can be appropriately modified. During the AV’s approach toward pedestrians, animation speed can be reduced; when the AV gets closer to pedestrians and deceleration is required, the animation pattern can further shift to the sweeping light. If the AV decides not to yield, it should initiate the light with fast animation speed so as to ensure the high intensity of visual signals. When the vehicle has already decided to yield to pedestrians, it should use lights with a gradually decreasing speed that matches the deceleration rhythm. To minimize pedestrians’ ambiguous perceptions of vehicle intent, designers should comprehensively consider the use of dynamic elements of the light-based eHMI based on specific conflict scenarios, traffic circumstance and current vehicle speeds, etc.

The objective results showed that participants held nearly balanced estimates about being allowed to cross the road when facing light-based eHMIs without motion cues (i.e., decelerating). When participants were asked to subjectively gauge the likelihood of the AV’s intent, they were slightly more inclined to report yielding. The difference between crossing decision and subjective evaluation reflected the fact that VRUs’ actual behavior would be more conservative. For practical design applications, we recommend lights serve as supplementary to text for the scenarios where a long-distance warning is necessary. It is reasonable to leverage characteristics of light-based eHMI to prompt early decisions, followed by explicit textual cues to prevent vehicle–pedestrian conflict. This could avoid pedestrians’ panic when reading text-based eHMIs under rapid approaching and eliminate the need for vehicles to reduce speed for their reading.

When using animation speed, the slow looping may be interpreted by pedestrians as indicating yielding, but a slow looping does not necessarily mean the vehicle is actively yielding. If the vehicle decelerates due to external road factors but its decision is not to yield, the light-based eHMI will still use a fast looping of animation. In this case, the light-based eHMI displays a warning while the vehicle is simultaneously slowing down. The phenomenon can be seen in Figure 8 where fast looping leads to more not-to-cross for a decelerating AV. For this scenario, we suggest adding a visual feature that indicates whether a pedestrian has been detected by the AV, because yielding is always targeted at the road user. If the AV is decelerating with fast looping of lighting animation but this feature is absent, it indicates that the AV has no intention of yielding. If the AV notices pedestrians but still does not yield, its autonomous driving style is extremely dangerous. In fact, a mature vehicle light design must integrate multiple visual features rather than using a single pattern.

### 4.2. Limitations and Future Work

A main limitation of this study in data statistics lies in the relatively small effect sizes. Under the same statistical power, sample size should be expanded to increase the effect sizes. Small effect sizes indicate that true differences between data groups of the factors and treatments are minor, meaning the impact of lighting characteristics as potential factors should not be overestimated. We only selected college students as participants, which may result in a certain degree of bias in estimating the overall effect. In contrast, the elderly population may require longer decision-making time due to longer observation time for light information. People with other social and cultural backgrounds may also respond differently to the eHMIs. Subsequent research should fully consider the differences in the subject population and supplement more data to enrich the current findings.

Regarding experimental design, this study has the following limitations. Firstly, only daytime scenarios where AVs communicate with the VRUs via light-based eHMIs were involved. At night, the light-based eHMI can be switched from constantly illuminated lights rather than suddenly activating as experimental stimuli. For example, the light representing non-yielding can become brighter than normal lights while those for yielding dim. Acting as the baseline, the impact of constantly illuminated lights on pedestrians’ predictions remains unclear. Moreover, we have not examined whether multimodal combinations of lighting characteristics outperform unimodal presentations. Nevertheless, we anticipate that amplifying or attenuating dynamic features conveys stronger signals than constant displays. Secondly, more types of vehicle light require investigation, particularly polychrome light and the headlights that project information onto the ground (in some designs the lights are projected from side-mounted sources of the car). The area of such a projection substantially exceeds vehicle-mounted displays, enabling more diverse indicative information with multiple colors. Understanding effective lighting features in projection designs remains lacking. Thirdly, the primary purpose of our study is to collect pedestrians’ decision biases based on personal traits and judgment criteria, instead of their instinctive behaviors when facing a real car. Therefore, we adopted video-based simulation, which cannot reproduce the pedestrian crossing scenarios involving environmental variables and the presence of other road users. To further validate the existing findings, real-world tests are still required to collect more objective user data.

Based on the current findings, follow-up studies must be conducted in the future to examine the interaction between the dynamic and static visual features of AVs’ lights, particularly the interaction of dynamic features with color. By observing the simple effects of different dynamic features and color, relatively effective combinations of lighting elements can be identified, which will enable a comprehensive evaluation of the applied value of all visual lighting elements. In the follow-up studies, various visual features can be integrated into representative light designs. Subsequently, field tests using physical cars should be conducted, with a substantial increase in sample size and demographic diversity. The testing methods may involve randomly presenting vehicles with different light features and observing participants’ one-time reactions to each feature.

## 5. Conclusions

This study thoroughly investigates the impact of dynamic features of light-based eHMIs on pedestrians’ crossing behavior, deriving several practically significant conclusions through systematic experimental design. Research reveals that animation speed plays a critical role in pedestrian decision-making. High speed of lighting animation significantly enhances pedestrians’ perception of non-yielding intent due to its potential warning effect, thereby effectively deterring crossing behaviors. In contrast, although animation patterns and light-emitting area influence decisions, their effects are comparatively weaker. The overarching implication of this study is that the specific elements of dynamic lighting eHMIs have an impact on pedestrians’ crossing behaviors. This study not only provides a valuable addition to the field of light-based eHMIs, but also offers important references for future designs for vehicle lights targeting warning or yielding intentions toward pedestrians in intelligent transportation systems. Designers can explore the optimal combination of dynamic features across diverse traffic scenarios and vehicle speeds to achieve more efficient and intuitive interaction of automated vehicles with the external road users.

## Figures and Tables

**Table 1:** The questionnaires for subjective ratings.

(**a**) Participants’ perceived likelihood that the AV detects the VRU and of vehicular yielding																
**Topic**	**Disagree**								**Agree**							
1. I think this vehicle light indicates that the AV has definitely detected my existence and appearance.	1	2		3		4	5		6		7	8		9		10
2. I think this vehicle light indicates a 100% likelihood of yielding of the AV.	1	2		3		4	5		6		7	8		9		10
(**b**) Task difficulty and perceived safety																
**Topic**	**Disagree**								**Agree**							
1. I feel it is not difficult for the pedestrian to make crossing decisions in this scene.	1		2		3			4		5			6		7	
2. I feel safe crossing the road in this scene.	1		2		3			4		5			6		7	

## Data Availability

The raw data supporting the conclusions of this article will be made available by the authors on request.
